# Combinatorial Engineering of 1-Deoxy-D-Xylulose 5-Phosphate Pathway Using Cross-Lapping *In Vitro* Assembly (CLIVA) Method

**DOI:** 10.1371/journal.pone.0079557

**Published:** 2013-11-05

**Authors:** Ruiyang Zou, Kang Zhou, Gregory Stephanopoulos, Heng Phon Too

**Affiliations:** 1 Chemical and Pharmaceutical Engineering, Singapore-MIT Alliance, Singapore, Singapore; 2 Department of Biochemistry, National University of Singapore, Singapore, Singapore; 3 Department of Chemical Engineering, Massachusetts Institute of Technology, Cambridge, Massachusetts, United States of America; Deutsches Krebsforschungszentrum, Germany

## Abstract

The ability to assemble multiple fragments of DNA into a plasmid in a single step is invaluable to studies in metabolic engineering and synthetic biology. Using phosphorothioate chemistry for high efficiency and site specific cleavage of sequences, a novel ligase independent cloning method (cross-lapping *in vitro* assembly, CLIVA) was systematically and rationally optimized in *E. coli*. A series of 16 constructs combinatorially expressing genes encoding enzymes in the 1-deoxy-D-xylulose 5-phosphate (DXP) pathway were assembled using multiple DNA modules. A plasmid (21.6 kb) containing 16 pathway genes, was successfully assembled from 7 modules with high efficiency (2.0 x 10^3^ cfu/ µg input DNA) within 2 days. Overexpressions of these constructs revealed the unanticipated inhibitory effects of certain combinations of genes on the production of amorphadiene. Interestingly, the inhibitory effects were correlated to the increase in the accumulation of intracellular methylerythritol cyclodiphosphate (MEC), an intermediate metabolite in the DXP pathway. The overexpression of the iron sulfur cluster operon was found to modestly increase the production of amorphadiene. This study demonstrated the utility of CLIVA in the assembly of multiple fragments of DNA into a plasmid which enabled the rapid exploration of biological pathways.

## Introduction

 Synthetic biology and metabolic engineering require convenient, robust and universal tools to manipulate genetic materials [1,2]. As such, a demand is to assemble multiple genetic components including sequences encoding enzymes, functional fusion tags and control elements (promoters, terminators and ribosome binding sites). The commonly used restriction enzymes and *in vitro* ligation based sequential cloning methods are often limited by the availability of unique restriction sites and are time consuming. Furthermore, single stranded DNA (ssDNA) overhangs generated by restriction enzymes are typically 2-8 nucleotides which exhibit poor annealing efficiencies and have limited use in assembling multiple large DNA fragments in a single step.

To address these challenges, several sequence independent methods, generating long ssDNA overhangs [3-13] or using double stranded PCR products with long homologous sequences [9,14,15], have been developed for the assembly of large DNA inserts into vectors. Only a few of these approaches have reported the assembly of multiple (>3) DNA fragments in a single step. Methods such as the T4 DNA polymerase based sequence and ligation-independent cloning (SLIC) [3], phosphorothioate-based ligase-independent gene cloning (PLICing) [13] and others [16-19] have only demonstrated the construction of plasmids of less than 8 kb. Various attempts have been made to meet the increasing demand to assemble several large fragments of DNA inserts into plasmids of > 10 kb [1,2,20]. A isothermal *in vitro* assembling method with synthetic oligonucleotides was used to assemble a 16.3 kb construct from seventy-five fragments of DNAs and the assembly of a 24kb plasmids from four separate fragments [9,21,22]. In addition, using yeast *in vivo* recombination system, a 582 kb *Mycoplasma genitalium* genome was constructed from synthetic DNA oligonucleotides in several steps [9]. The yeast system has also been successfully used for the one step assembly of a 19kb fragments into a plasmid or yeast chromosome [15]. With these examples, homologous overhang sequences with lengths of 100-500 base pairs were required to increase the assembly efficiency. This can be a significant challenge where suitable pre-existing sequences in the parental or chemically synthesized templates are required which can restrict the applicability and incur high-cost of synthesis. Furthermore, these approaches are also time consuming and labor intensive, hence, are not suited for routine cloning projects. 

Here we report the development of a reliable, scalable and robust cloning method (cross-lapping *in vitro* assembly, CLIVA) for the rapid construction of large recombinant DNA from multiple fragments in a single step. This approach exploits the unique properties of phosphorothioate modified nucleotides where highly efficient and site specific cleavage is achieved using iodine in an ethanolic solution [23,24]. Recently, Milan Blanusa et al [12] demonstrated the use of such phosphorothioate chemistry for the assembly of multiple small protein domains [13]. Unique to the CLIVA method is a novel cross-lapping design which allows the generation of long homologous overhang sequences (36-38 bases) by cleavage of optimally positioned phosphorothioate modified nucleotides and the use of selective cations resulting in a highly efficient assembling process. To demonstrate the utility of this method, we constructed 16 plasmids of 7.8 kb to 21.6 kb in size, encoding various combinations of genes in the 1-Deoxy-D-xylulose 5-phosphate (DXP) pathway in *E. coli*. To our knowledge, this is the first report of the successful assembly of large constructs containing multiple genes using an enzyme independent *in vitro* method to engineer multi-enzyme pathways in a short duration.

Isoprenoids are a large and diverse class of natural products (more than 55,000) derived from five-carbon isoprene units. Some are fragrances, insecticides, nutraceuticals and pharmaceuticals [25], while the functions of the vast majority of the isoprenoids remain to be determined [26]. Due to the structural complexities of many of these compounds, e.g., Artemisinin [25] and Taxol [26], *de novo* total chemical synthesis is impractical. Metabolic engineering of microbes is a promising alternative and has been intensively explored by manipulating the 1-deoxy-D-xylulose-5-phosphate (DXP) [27] or the mevalonate (MVA) pathway [28]. The DXP pathway displays a more balanced redox utility as compared to the MVA pathway *in vivo* [29]. In *E. coli*, a few empirically selected enzymes (*dxs*, *idi*, *ispD*, *ispF*) are thought to be the limiting steps in the DXP pathway and increasing the expression levels of these enzymes have been shown to improve isoprenoid production [29-33]. 

**Figure 1:**
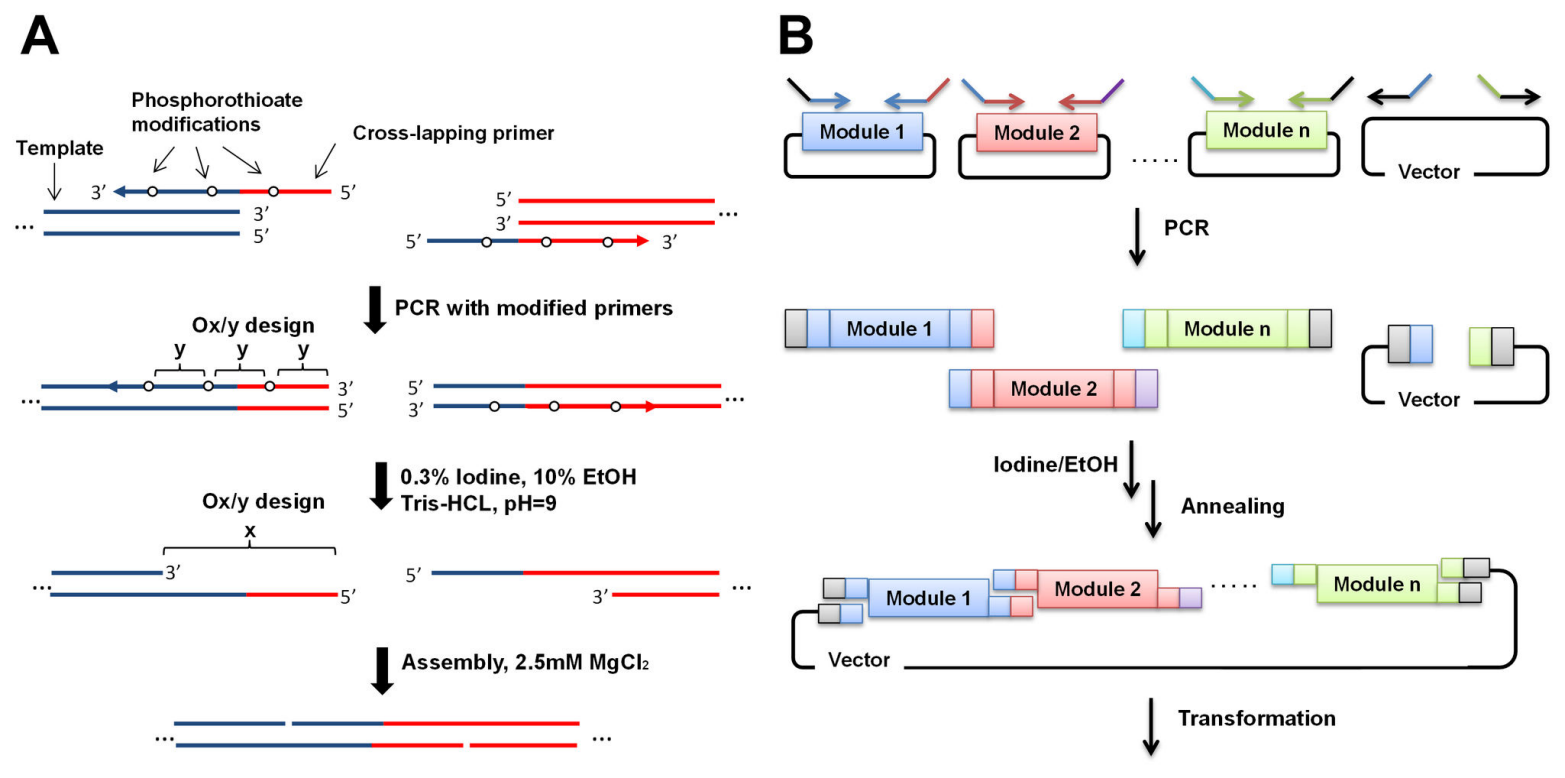
Assembly of DXP pathway. (A) The dxp pathway and Fe-S cluster assembling pathway. GA3P: glyceraldehyde 3-phosphate, DXP: 1-deoxy-D-xylulose 5-phosphate, MEP: 2C-methyl-D-erythritol 4-phosphate, CDP-ME: 4-diphosphocytidyl-2C-methyl D-erythritol, CDP-MEP: 4-diphosphocytidyl-2C-methyl D-erythritol 2-phosphate, MEC: 2C-methyl-D-erythritol 2,4-diphosphate, HMBPP: hydroxylmethylbutenyl diphosphate, IPP: Isopentenyl pyrophosphate, DMAPP: Dimethylallyl pyrophosphate, GPP: Geranyl diphosphate, FPP: Farnesyl diphosphate, GGPP: Geranylgeranyl diphosphate. (B) Illustration of various modules assembled in the project (correlated to Table S3). CAM: chloramphenicol resistance gene, p15A-ori: p15A original of replication.

In this study, the effects of various combinations of the enzymes in the DXP pathway in providing precursors to downstream production of amorphadiene, the precursor for antimalarial drug artemisinin [30], was systematically investigated for the first time (Figure 1A). The CLIVA method enabled the assembly of multiple plasmids containing various combinations of genes rapidly. Metabolic profiling using ultra-performance liquid chromatography mass spectrometry (UPLC-MS) [31] identified the accumulation of intracellular MEC (one of the DXP pathway intermediate) as a limiting factor for isoprenoid production. The overexpression of iron sulfur cluster (Isc) operon, which supplied the cofactors for the function of two succeeding enzymes downstream of MEC (*ispG* and *ispH*) (Figure 1A), was found to modestly enhance the production of amorphadiene.

## Results

### Design of CLIVA

**Figure 2:**
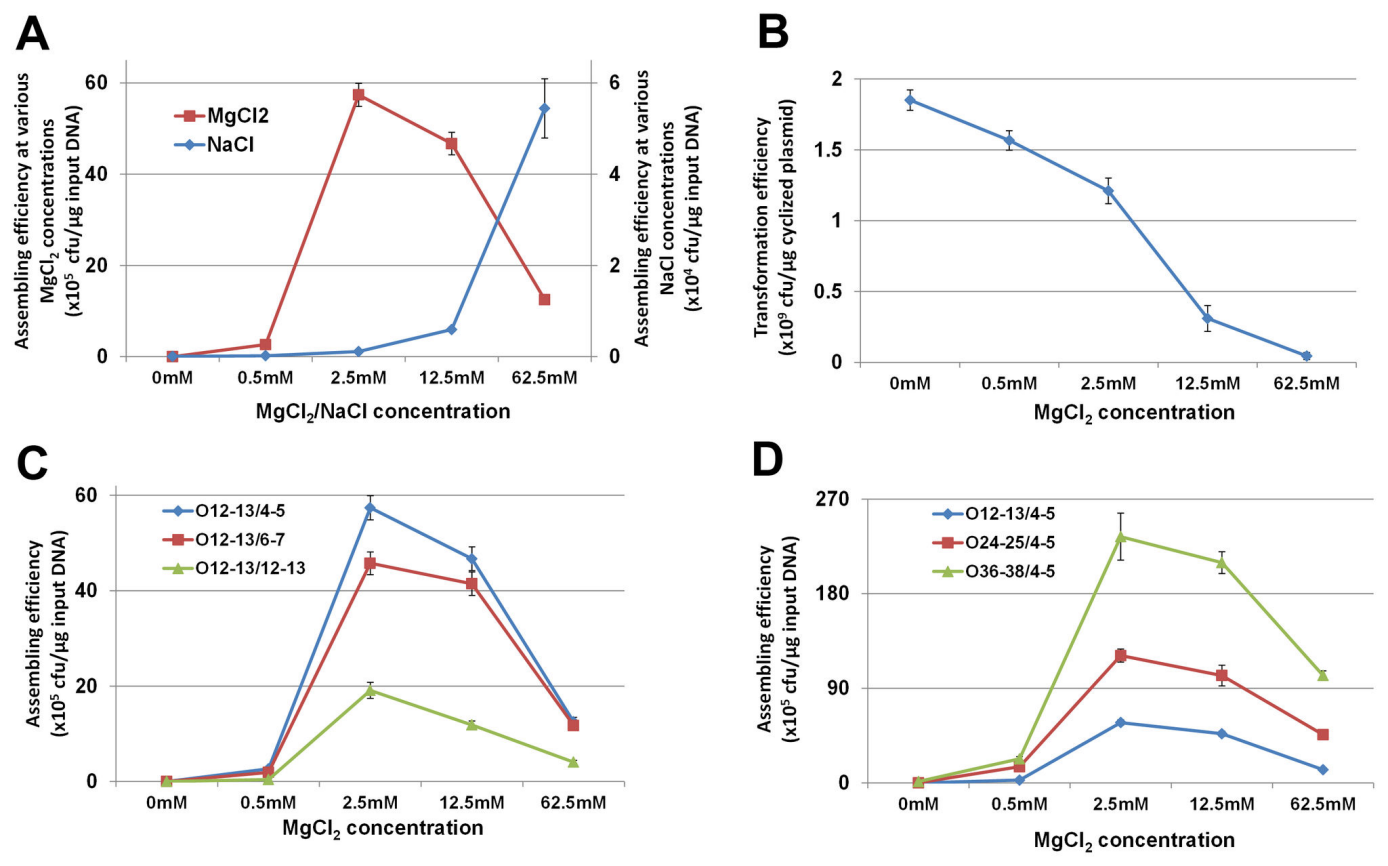
The cross-lapping in vitro assembly (CLIVA) method. (A) Illustration of the design at one junction between two modules (blue and red). The cross-lapping primer consists of gene specific sequence (GSS) and tag sequence complementary to adjacent primer’s GSS. The phosphorothioate modifications were indicated as cycles. An “Ox/y” designation was used to define the primers, where O denoted overlap; x was the length of overlap which had one modification at each y base pairs of the sequence. (B) Illustration of assembling of multiple DNA modules into one plasmid.

PCR has been used to produce overlapping homologous sequences by adding extraneous tag sequences to the gene specific primers [3,12,15]. With such a design, the homologous sequences are limited to the length of the tags. In order to increase the assembly efficiency, we designed the tags to be homologous to the gene specific sequences (Figure 1A). This cross-lapping design allowed us to increase the length of the homologous sequences at each junction as compared to conventional strategies. Besides, other than modifying all the bases in the homologous sequences which increased the cost of primer synthesis, we explored the possibility of decreasing the modification frequency (number of phosphothiodate modification per oligonucleotide) while maintaining a high efficiency of assembly (Figure 2A). Critically, by the use of certain cations, the efficiency of the assembly process was substantially increased and this has enabled the construction of large plasmids from multiple fragments in one step. 

In order to demonstrate the utility of this method, we constructed a series of plasmids carrying multiple genes of a metabolic pathway. As shown in Figure 2B, all the pathway modules as well as a vector module containing the origin of replication and antibiotic resistant gene were first amplified from the parental plasmids using a pair of cross-lapping primers and subsequently treated with a solution of ethanolic iodine as described in “MATERIAL AND METHODS”. The assembly was then carried out in the optimal condition with equal molar of each DNA module fragment (see below). 

### Optimization of CLIVA

The construction of a 7.1 kb PAC-SIDF plasmid was initially used as a model for identifying suitable designs and optimal conditions for CLIVA. The PAC-SIDF plasmid was generated by combining two modules amplified from different sources: the PAC vector (2.8 kb) consisting of P15A origin of replication and chloramphenicol resistant gene (Figure 1B) from a pre-existing pAC-lyc plasmid [32] and SIDF module (4.3 kb) containing four 1-Deoxy-D-xylulose 5-phosphate (DXP) pathway enzymes (*dxs*, *idi*, *ispD*, *ispF*, Figure 1A) from a pre-existing pET-dxs-idi-ispDF plasmid [33]. All the primers used in the optimization process were listed in Table S1 where the PAC-F/PAC-R and SIDF-F/SIDF-R were the gene specific sequences targeting at pAC-lyc plasmid and pET-dxs-idi-ispDF plasmid. 

**Figure 3:**
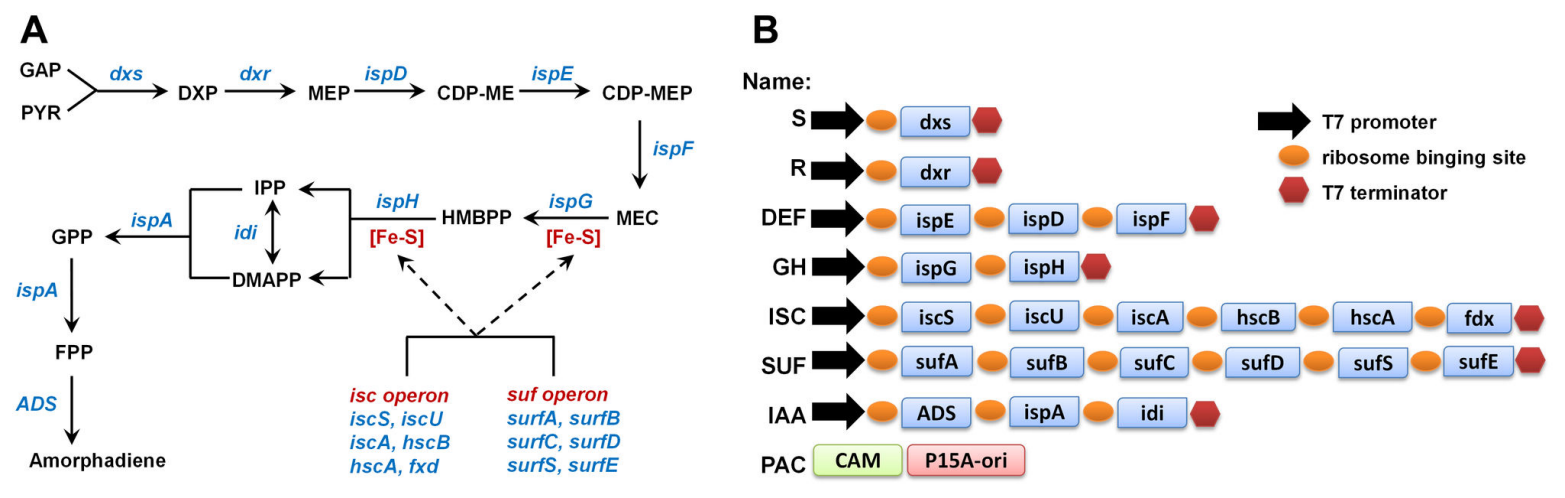
Optimization of CLIVA method. (**A**) Optimization of cations using the assembly of PAC-SIDF plasmid with O12-13/4-5 design (12-13 bases overlap with modification at every 4-5 bases). (**B**) The transformation efficiency of PAC-SIDF plasmid in the presence of MgCl_2_. (**C**) The effect of the phosphorothioate modification frequency on the assembly efficiency. O12-13/4-5, O12-13/6-7, O12-13/12-13 designs: 12-13 bases overlap with modification at every 4-5 bases, 6-7 bases or 12-13 bases. (**D**) The effect of overlap length on the assembly efficiency. O12-13/4-5, O24-25/4-5, O36-38/4-5 designs: 12-13 bases, 24-25 bases, 36-38 bases overlap with modification at every 4-5 bases. All the experiments were done at triplicates and the standard errors were shown in the figure.

Ionic strength is known to affect DNA hybridization [34]. As cations can reduce charge repulsion between the negatively charged phosphodiester backbones of double stranded DNA, we sought to investigate the assembly efficiency in relation to the concentrations of MgCl_2_ or NaCl. The assembly efficiency increased dramatically with the addition of salts and the divalent cation (Mg^2+^) resulted in much higher enhancement (Figure 3A), consistent with the findings of others [35]. With respect to Na^+^, there was a positive correlation between the ionic concentration and the assembly efficiency. With Mg^2+^, a decrease in the assembly efficiency was observed at high concentrations. A limitation in using high concentrations of salts (NaCl or MgCl_2_) was that these reaction mixtures were incompatible with the use of electroporation for transformation. This proposal was consistent with the observation of the severe suppression of transformation efficiency at high MgCl_2_ concentration (62.5 mM) (Figure 3B). Thus, the optimum MgCl_2_ concentration was identified as 2.5mM. We also tested other divalent ions (CuCl_2_, CaCl_2_, and CoCl_2_) and found that Ca^2+^ acted similarly to Mg^2+^, while Co^2+^ and Cu^2+^ were found to be significantly poorer (Figure S1). This was possibly due to the toxicity of Co^2+^ and Cu^2+^ ions at high concentrations [36,37]. 

Existing methods that generate ssDNA with phosphorothioate chemistry have every base of the overlap sequence chemically modified, which is cost prohibitive for long overlapping sequences [12,13]. We hypothesized that it was unnecessary to cleave the overlapping sequence into single bases; instead, by cleaving the nucleotide at several discrete sites into smaller fragments, the assembly should work equally well. We then tested this hypothesis using four types of 12-13 bases overlap designs: O12-13/1, O12-13/4-5, O12-13/6-7 and O12-13/12-13 with different positions of the sequences modified with phosphorothioate where the modifications at positions were 1 base apart, 4-5 bases apart, 6-7 bases apart or 12-13 bases apart, respectively (Table S1). Unexpectedly, amplification using O12-13/1 primer pairs (modification inserted at every base) yielded extremely low amount of amplicon and was not used for further studies. The exact reason for this poor amplification is currently unknown. Nonetheless, the O12-13/4-5 design was successfully amplified showed a high assembly efficiency. A slightly lower assembly efficiency was observed when using the O12-13/6-7 design and even lesser still with the O12-13/12-13 design (Figure 3C). It is worthy to note that with the O12-13/12-13 design where a single modification was incorporated, the cleavage resulted in a fragment of the DNA which was identical to the overlap sequence and hence, may have competed for annealing. So this arrangement would result in a lower efficiency in assembly, consistent with the observation in Figure 3C. Increasing the modification frequency greater than one in 4-5 bases apart did not substantially improve the efficiency of assembly as compared to one in 6-7 bases. 

Another critical parameter for the assembly of multiple DNA fragments is the length of the overlaps that determines the specificity as well as the efficiency of the annealing. As predicted, when compared to short overlaps (12-13 bases), the assembly efficiency increased with longer overlapping segments (36-38 bases) by as much as 3 fold (Figure 3D). With the increasing number of pathway modules to assemble, it is critical to have high assembly efficiency at each junction

Extending the study, the assembling efficiencies of designs with only a single phosphorothioate modification (O12-13/12-13, O24-25/24-25 and O36-38/36-38) were examined (Figure S2). With this arrangement, the design with longer overlap sequences after cleavage (O24-25/24-25 where the overlap was 24-25 bases) showed lower efficiency of assembly than a shorter one (O12-13/12-13 where the overlap was 12-13 bases). In addition, an even longer overlap (the O36-38/36-38 design where the overlap was 36-38 bases) was even poorer. Thus, with single phosphorothioate modification, the efficiency of assembly was related to the length of the cleaved product whereby the fragmented pieces of DNA should be short so as not to interact with the overlap sequences. Thus, the O36-38/4-5 design was suitable for the assembly of multi-components with high efficiency, while the O12-13/12-13 design was sufficiently efficient and cost effective, replacing the use of restriction enzyme and ligation based method for routine tasks.

### Constructions of plasmids using CLIVA method

Next, we used the CLIVA method to assemble a series of plasmids consisting of various combinations of modules containing the genes of the 1-Deoxy-D-xylulose 5-phosphate (DXP) pathway [27] and for amorphadiene production (Figure 1A). In addition, two operons, ISC (iron-sulfur cluster (Isc) operon) and SUF (sulfur mobilization (Suf) operon), containing the proteins necessary for Fe–S cluster [38] assembly in *E. coli* were also constructed (Figure 1A). Details of the modules and their abbreviations were presented in Figure 1B. Fragments of treated DNAs were mixed and transformed into *E. coli* for the one step assembly of these genes (Figure 2B) and the correct clones were identified by quantitative colony PCR as described in “MATERIAL AND METHODS”. With each construct, two randomly selected positive clones were further confirmed by restriction mapping and at least one of these was verified by sequencing. The sequencing results covered all the sequences encoding the junctions (the overlap sequence between the modules) as well as more than 50% of the sequences in the plasmid. No change in the sequences was observed, indicative of the high fidelity of amplification and high specificity of cleavage. As expected, the efficiency decreased with increasing number of fragments (Table 1). However, even with the largest plasmid (21.6kb, S-R-DEF-GH-ISC-IAA-PAC plasmid from 6 modules) assembled, the efficiency was reliably high (~2.0 x 10^3^ cfu/μg input DNA). The false positive colonies resulting in lower accuracy of assembly were largely due to the existence of plasmids with incomplete pathway modules (demonstrated by quantitative colony PCR and restriction mapping, data not shown).

**Table 1:** Construction efficiency of the DXP pathway plasmids using CLIVA method.

**Plasmids**	**Size (kb)**	**Number of pieces to assemble**	**Transformation efficiency (x10^3^ cfu/µg input DNA)**	Accuracy (%)*
**IAA-PAC**	6.2	2	3612.8	100.0
**S-IAA-PAC**	8.7	2	1052.8	100.0
**S-R-IAA-PAC**	10.5	3	78.8	93.5
**S-DEF-IAA-PAC**	11.3	3	61.3	96.8
**S-GH-IAA-PAC**	11.3	3	46.6	83.9
**S-R-DEF-IAA-PAC**	13.1	4	13.2	42.6
**S-R-GH-IAA-PAC**	13.1	4	15.6	38.3
**S-DEF-GH-IAA-PAC**	13.9	4	9.0	27.7
**S-R-DEF-GH-IAA-PAC**	15.6	5	5.1	12.7
**S-ISC-IAA-PAC**	14.2	3	15.4	25.5
**S-SUR-IAA-PAC**	14.7	3	17.4	21.3
**S-GH-ISC-IAA-PAC**	16.8	4	4.9	14.1
**S-GH-SUR-IAA-PAC**	17.2	4	4.5	11.3
**S-R-GH-ISC-IAA-PAC**	18.5	5	3.1	9.9
**S-R-GH-SUR-IAA-PAC**	19.0	5	2.6	7.0
**S-R-DEF-GH-ISC-IAA-PAC**	21.6	6	2.0	8.5

* More than 30 colonies for each construct were analyzed by quantitative colony PCR for the accuracy calculation.

### Overexpression of GH and R-DEF inhibited amorphadiene production

**Figure 4:**
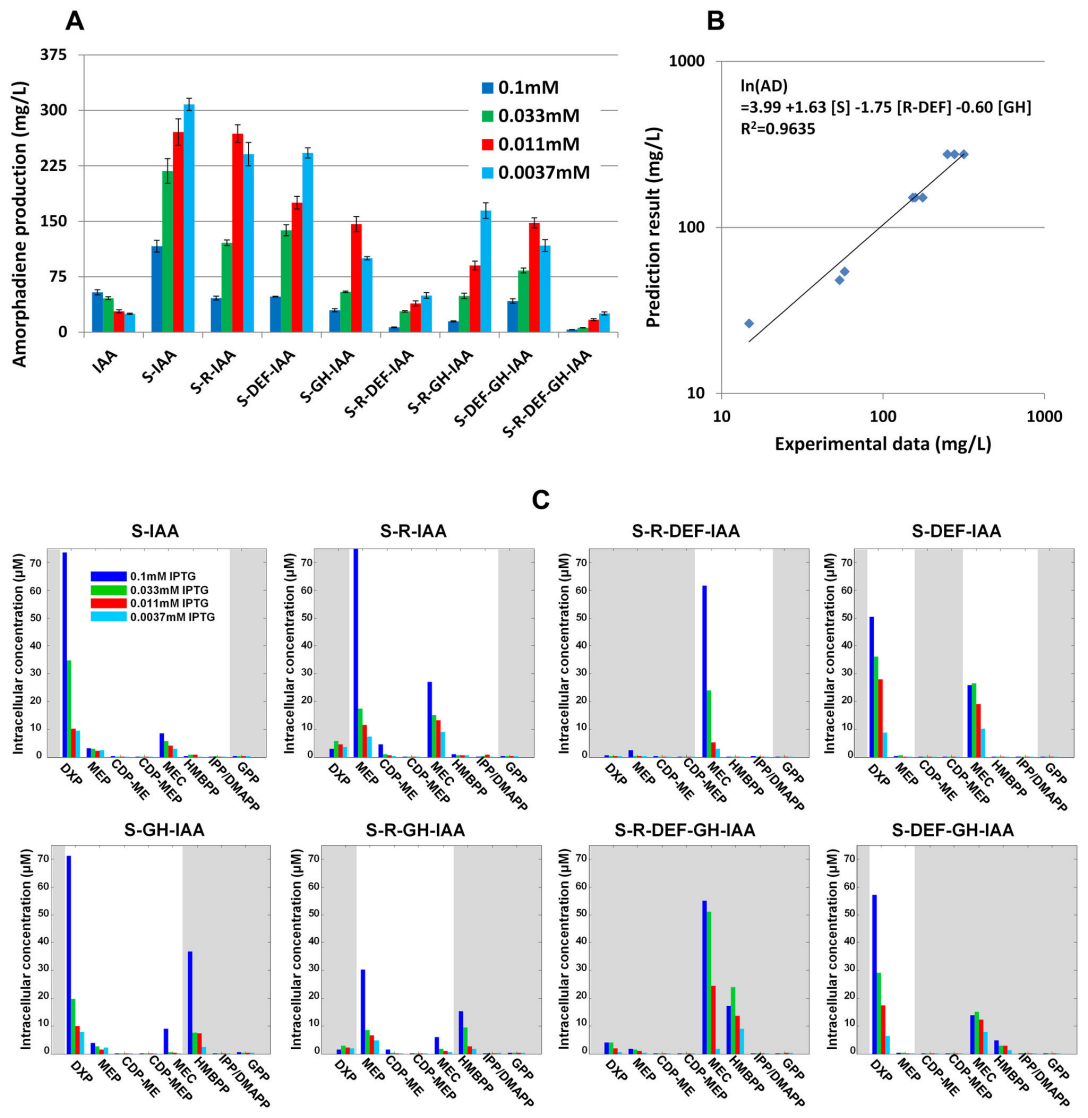
The performance of different combinations of DXP pathway genes in *E*. *coli*. (A) 48h amorphadiene yield. Different concentrations of IPTG were represented by bars with different colors. The experiment was repeated four times and the standard errors were shown. (B) The correlation of pathway modules with amorphadiene yield at optimal IPTG inductions. (C) Early response of intracellular metabolites at 3h after induction. The gray areas indicated the overexpressed section of DXP pathway. The experiment was repeated twice and the averages were shown.

Next, the various combinations of pathway genes with the essential module (IAA) containing the heterologous amorphadiene synthase were tested for amorphadiene production. Consistent with previous reports [31,39,40], high induction resulted in lower production of isoprenoids (Figure 4A, different IPTG inductions). Comparing constructs at their optimal induction levels, as expected, the expression of the first committed step (*dxs* – module S) enhanced the amorphadiene production. However, the overexpression of the rest of the pathway genes in conjunction with the S and IAA modules had variable negative effects on productivity. Notably, the expression of GH module (*ispG* and *ispH*) as well as R (*dxr*) -DEF (*ispD*, *ispE* and *ispF*) modules led to a significant inhibition on the production (Figure 4A). Consistent with the observations, a simple linear model correlating the pathway modules and amorphadiene yields at their optimal inductions revealed that the expression of GH module or the co-expression of R-DEF modules had negative impacts (Figure 4B). 

In order to investigate the changes in the levels of intracellular metabolic intermediates with the overexpression of the various modules, cells were harvested after 3 h of induction and the metabolites were quantified by UPLC-MS (Figure 4C). The induction of the expression of the genes in any of the modules resulted in significant accumulation of intracellular MEC, indicative of a limitation in metabolite conversion with genes downstream, an observation in congruence with our previous report [31]. Interestingly, the overexpression of GH module did not fully convert MEC to the downstream metabolite IPP/DMAPP. Instead the metabolite HMBPP accumulated in all strains where the GH module was overexpressed (Figure 4C, the second row). Other than that, the genes in the pathway upstream of MEC were functionally expressed as the accumulations of the metabolites were positively correlated with the expressed genes. Hence, the overexpression of *dxs*, the first and committed step in the DXP pathway, resulted in the accumulation of DXP (Figure 4C, S-IAA). Similarly, the overexpression of *dxs* and *dxr* resulted in the accumulation of MEP (Figure 4C, S-R-IAA) and the co-expression of S-R-DEF resulted in the high accumulation of MEC (Figure 4C, S-R-DEF-IAA). Besides, higher expressions of these genes resulted in the parallel increases in activities (higher concentrations of accumulated intermediates). 

### Accumulation of intracellular MEC was inversely correlated to amorphadiene productivity

**Figure 5:**
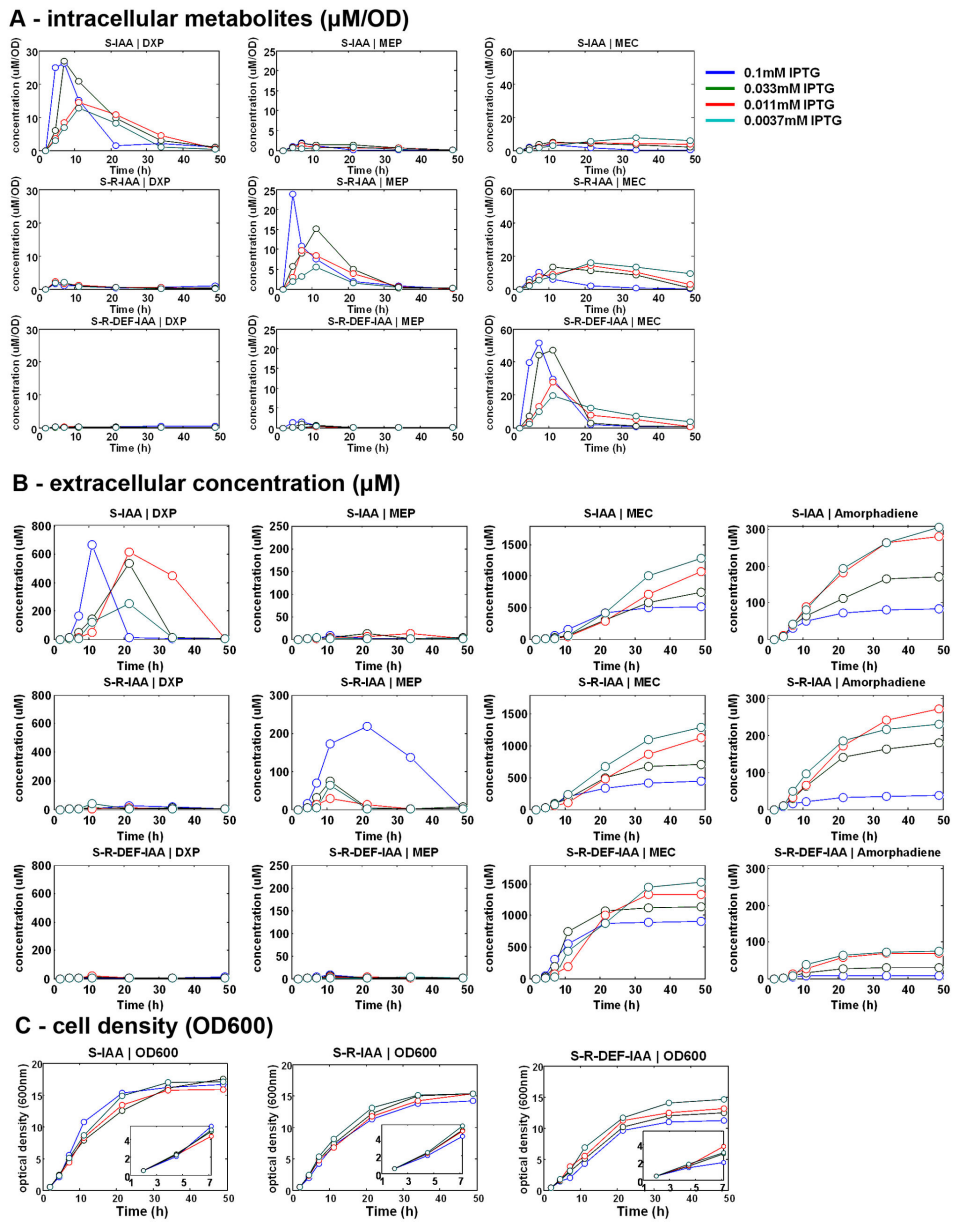
The kinetics of S-IAA-PAC, S-R-IAA-PAC and S-R-DEF-IAA-PAC strains. (**A**) The specific concentration (μM/OD) of intracellular metabolites: DXP, MEP and MEC. The rest of the metabolites were accumulated at concentrations lesser than 2µM/OD and were neglected. (**B**) The concentration of extracellular metabolites: DXP, MEP, MEC and amorphadiene. The rest of the metabolites were accumulated at concentrations lesser than 50µM and were neglected. (**C**) The cell density.

In order to further investigate the pathway, a kinetic study measuring the concentrations of intracellular, extracellular DXP metabolites and amorphadiene was carried out with strains harboring different modules. As expected, the induction of *dxs* resulted in a significant increase in the level of intracellular DXP in the strain with S-IAA modules (Figure 5A, S-IAA|DXP). Curiously, extracellular level of DXP was also increased substantially albeit with different kinetics (Figure 5B, S-IAA|DXP). Similarly, the expression of the S-R-IAA modules resulted in the accumulation of both intracellular and extracellular MEP (Figure 5A, B S-R-IAA|MEP). With all three modules, MEC accumulated intracellularly and significantly more with the S-R-DEF-IAA modules. Intriguingly, the extracellular levels of MEC accumulated to similar levels and were inversely correlated to the inducer concentrations in strains carrying any of the three modules (Figure 5B, MEC). The inverse correlation of metabolite levels with the inducer concentration used was also observed with the production of amorphodiene. The S-R-DEF-IAA-PAC strain accumulated large quantities of intracellular MEC and yielded much less amorphodiene as compared to strains harboring the S-IAA or S-R-DEF-IAA modules (Figure 5B, MEC). Although high IPTG inductions yielded higher concentrations of intracellular intermediates initially (Figure 5A, first 10 h), the relationship was reversed at later time points, especially with the highest induction (0.1mM IPTG) (Figure 5A, highly accumulated intermediates). Other metabolites (CDP-ME, IPP/DMAPP, GPP, FPP) were found to be accumulated at insignificant levels. 

### Overexpression of Fe–S operons modestly increased amorphodiene productivity

**Figure 6:**
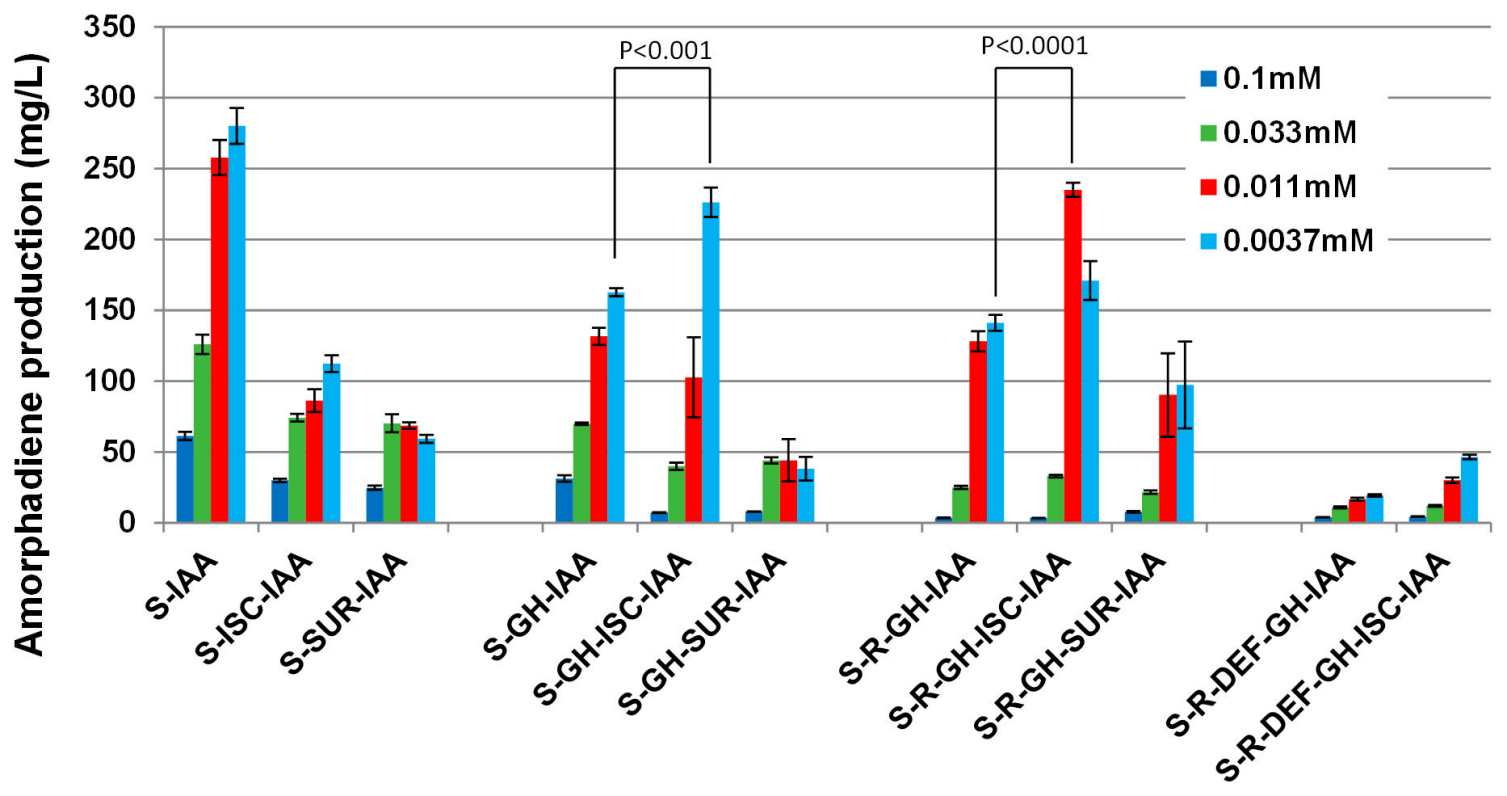
The effects of Fe-S operons on the amorphadiene production. Different concentrations of IPTG were represented by bars with different colors. The experiment was repeated four times and the standard errors of four replicates were presented as error bars. The two tailed p-values of student’s t-test were carried out to compare certain conditions and presented as P in the figure.

An attempt was made to increase the activities of *ispG* and *ispH* (GH module) in converting MEC to the downstream metabolite IPP/DMAPP so as to increase amorphodiene production. As the essential cofactor for these two enzymes, the genes in the iron-sulfur (Fe-S) cluster pathways (iron-sulfur cluster (Isc) operon- *iscS*, *isCU*, *iscA*, *hscB*, *hscA*, *fdx*) and/or sulphur mobilization (Suf) operon (SUF module (*surA*, *surB*, *surC*, *surD*, *surS*, *surE*) [38,41] were assembled using CLIVA and transformed into *E. coli*. Disappointingly, the overexpression of either operon together with S-IAA modules not only did not enhance but instead inhibited the production of amorphodiene (Figure 6, 1-3 columns). The overexpression of Isc operon in other constructs together with GH module showed modest enhancements (Figure 6, 4-8 columns).

## Discussion

This study demonstrated the rapid assembly of large plasmids with an array of metabolic genes (21.6 kb plasmid with 16 genes) using a ligation independent cloning (CLIVA) method. These recombinant plasmids were then used to systematically investigate the effects of the various combinations of the enzymes in the DXP pathway in producing amorphadiene, the precursor for antimalarial drug artemisinin [30] (Figure 1A). Metabolic profiling using ultra-performance liquid chromatography mass spectrometry (UPLC-MS) [31] identified the accumulation of intracellular MEC (one of the DXP pathway intermediate) as a potential negative contributor to isoprenoid production. The overexpression of the Isc operon, which supplied the cofactor for the function of two succeeding enzymes downstream of MEC (*ispG* and *ispH*) (Figure 1A), was found to modestly increased the production of amorphadiene.

The manipulation of genetic material is a fundamental and routine requirement for engineering of biological systems where multiple genes are assembled and used to produce downstream products [1,2]. The traditional *in vitro* ligation based cloning methods are sequence-dependent and are often not efficient in assembling multiple fragments of DNAs. Consequently, these limitations have been addressed with methods that assemble multiple DNA fragments with overlapping homologous sequences in a single step. Such *in vitro* assembling method [9,21,22] or the yeast *in vivo* homolog recombination based DNA assembler method [15] uses enzymes with exonuclease activities to generate ssDNA and other enzymes to repair the over-treated non-homologous ssDNA gaps. The use of multiple enzymes does not only incur cost but is also inefficient and time consuming. Based on the phosphorothioate chemistry that allows cleavage of DNA at specific sites, the enzyme-free CLIVA method provides robust performance for the one-step assembly of multiple DNA modules. Typically, the construction can be completed within 1-2 days, as compared to the more involved method of yeast recombination (1-2 weeks). 

The novel design of the cross-lapping PCR primer pair (~40 bases) enabled high efficiency of amplification by PCR and efficient assembly of multiple DNA fragments. Unlike other studies [12,13], we found that phosphothioate modifications of every 4-5 bases intervals in the homologous sequences was sufficient to enable efficient cleave and assembly of the sequences. The use of cations at optimal concentration was found to significantly enhance the assembly efficiency while maintaining high transformation efficiency. Even with a single phosphothioate modification, the assembly of two pieces of DNA fragments (~3-4 kb each) was highly efficient (~ 2.0x10^6^ cfu/ μg input DNA). This was far superior to the use of restriction enzymes and ligase (< 10^4^ cfu/ μg input DNA for the same construct) in parallel studies. Hence, the CLIVA method can replace all routine recombinant DNA constructions with the use of just a single phosphothioate modification in each primer. The assembly of the 21.6kb plasmid (S-R-DEF-GH-ISC-IAA-PAC) from 6 fragments of DNAs was sufficiently efficient (~2.0 x 10^3^ cfu/μg input DNA) and was completed in less than 2 days. 

With constructs encoding multiple genes under the control of the same regulatory elements (T7 promoters and terminators), there were large amount of repeated sequences (200-300 bps) in regions between modules. As those perfect repeats may randomly anneal with each other during assembly, it was not surprising that the assembly of such multiple identical sequences resulted in numerous false positive clones which contained partially assembled sequences, an observation confirmed by quantitative colony PCR and restriction analysis. The use of the same regulatory elements to control multiple modules is predictably to be even more challenging for recombination based methods which are known to selectively rearrange repeated sequences *in vivo* [15]. 

The S-R-DEF-IAA-PAC strain resulted in lesser yield of amorphadiene as compared to the other strains (S-IAA-PAC or S-R-IAA-PAC) which encode fewer numbers of genes in the pathway. The overexpressions of this poor performing construct resulted in transient accumulations of high levels of intracellular MEC but yet showed similar extracellular levels with the other modules. The inverse relationship of the levels of intracellular MEC and the downstream metabolite productivity suggests an inhibitory role of MEC in regulating isoprenoid production, possibly due to the increase in oxidative stress in the cell [42-44]. Recently, MEC was also identified as a signaling molecule that induces stress-responsive genes in plant [45], consistent with an involvement in stress response. Whether such stress response mechanism occurs in these strains remains to be determined.

The overexpression of module (GH) containing *ispG* and *ispH* resulted in the accumulation of HMBPP and yet did not increase amorphodiene production as would have been anticipated. A possibility is the limitation in the co-factor system [38,41] which involved the iron-sulfur cluster an observation consistent with a recent report in *S. cerevisiae* [46]. The co-expression of Isc operon did enhance the production of amorphadiene production but the yield was significantly lower than in strain overexpressing the S-IAA modules. Although only modest enhancement was observed when the GH module was co-expressed with ISC module, fine tuning of those genes (*ispG*, *ispH*, *iscS*, *isCU*, *iscA*, *hscB*, *hscA*, *fdx*) including controlling the expression levels and additional combinations can be further explored to increase the flux of intracellular MEC.

Given the need to construct multiple vectors, the CLIVA method described herein provides a rapid, effective and efficient approach to identify combinations of genes useful for the production of metabolites. In this study, we found that the overexpression of related pathway genes may not simply enhance but may unpredictably inhibit downstream metabolite production. Given the complexity of cellular regulatory pathways and experimental conditions, a systematic approach to identify optimal combinations of genes for high yield production will necessitate the construction of arrays of recombinant plasmids using the CLIVA method described herein. 

## Materials and Methods

### Reagents, growth medium and bacteria strain

Restriction enzymes were purchased from NEB. The high fidelity DNA polymerase (iProof^TM^) from Bio-Rad was used to amplify the DNA fragments for assembly and the iTaq^TM^ DNA polymerase from iDNA was used for quantitative colony PCR. Unless stated otherwise, all chemicals were purchased from either Sigma or Merck. Peptone and yeast extract were purchased from BD. Oligonucleotides were purchased from AITbiotech. Unmodified oligonucleotides were purified by desalting and the phosphorothioate modified oligonucleotides were purified with cartridge. All the cells for plasmid construction were grown in 2xPY media or 2xPY agar plates containing: peptone (20 g/L), yeast extract (10 g/L) and NaCl (10 g/L) with or without agar (7.5 g/L). The *E. coli* XL10-Gold strain (Invitrogen) was used for plasmid construction. The electroporation competent cells were prepared: 1 L of XL10-Gold cells at OD600~= 0.4, washed for three time with equal volume of 10% cold glycerol, suspended in 10 ml of cold 10% glycerol and stored at -80 °C. For amorphadiene production, the *E. coli* Bl21-Gold DE3 strain (Stratagene) harboring different kinds of DXP pathway plasmid together with the pRepressor plasmid carrying the lac repressor gene was cultured in production medium: peptone 20 g/L, yeast extract 10 g/L, NaCl 10 g/L, glycerol 20 g/L, HEPES 50 mM and Tween 80 5 g/L. The pRepressor plasmid was constructed by removing the T7 promoter, RBS and T7 terminator of pET-11a (Stratagene) plasmid and replacing the antibiotic resistant (ampicillin) with kanamycin. All the culture contained 34 mg/L chloramphenicol and 100 mg/L kanamycin to maintain the DXP pathway plasmid and pRepressor plasmid respectively. The cell density was defined by absorbance at 600 nm (OD600) and measured by SpectraMax 190 microplate reader. For amorphadiene production, 1% (v/v) cell culture of overnight grown cell culture was inoculated into 0.8ml production medium together with another 0.2 ml organic dodecane phase to extract amorphadiene in 14mL BD Falcon^TM^ tube. The dodecane phase contained 1 g/L trans-caryophyllene as internal standard for amorphadiene. Cells were grown at 37 °C with 300 rpm shaking for 2 h when OD600 reached the range of 0.5 - 0.8 and induced by different concentrations of isopropyl β-D-1-thiogalactopyranoside (IPTG). After induction, the cell was incubated at 28 °C with 300 rpm shaking for the rest of the experiment. The induction time was considered as the zeros time point in the study. 

### Quantitative colony PCR

The quantitative colony PCR was carried out to test the presence of successful ligations at all the junctions of constructed plasmids using the primer listed in Table S4. For example, to confirm the S-GH-IAA-PAC plasmid, the junctions of PAC-S, S-GH and GH-IAA were verified by quantitative colony PCR respectively. For each junction, the sense primer in the upstream module and antisense primer in the downstream module were used as a pair to perform the real-time quantitative PCR, which were dxs-1609F/ispG-329R, ispH-693F/ADS-941R and PAC-seqF/dxs-122R pairs respectively. For quantitative colony PCR, the overnight cultured colonies were suspended in 100 µl of water. The real-time quantitative PCR reactions were carried out in 25 µl final volume containing 5 µl of cell suspension, 1x Xtensa Buffer (Bioworks), 200 nM of each primer, 2.5 mM MgCl_2_ and 0.75 U of iTaq DNA polymerase (iDNA). The reactions were analyzed using a BioRad iCycler 4^TM^ Real-Time PCR Detection System (Bio-Rad) with SYBR Green I detection and the following protocol: an initial denaturation of 10 min at 95 °C to lyse the cells, followed by 40 cycles of 30 s at 95 °C, 30 s at 60 °C, and 1 min at 72 °C. A melt curve was then carried out to check the melting temperature of the amplicon. Various primer pairs were selected from Table S4 to measure different module linkages in all the selected colonies. The results with a Ct number earlier than 18 and correct melting temperature were recognized as positive. 

### Plasmid assembling by CLIVA method

The primers for CLIVA optimization studies are listed in Table S1 and for DXP pathway assembling are listed in Table S2. The design details for all the 16 constructed plasmids are listed in Table S3. The modules containing various DXP pathway genes (*dxs*, *dxr*, *ispD*, *ispE*, *ispF*, *ispG*, *ispG*, *idi*, *ispA* or iron-sulfur (Fe-S) biosynthesis pathway (Isc operon, Suf operon), Figure 1B) were amplified from the source plasmids constructed by placing those genes between T7 promoter and T7 terminator in pET-11a plasmid from Stratagene. The genomic DNA purified from MG1655 DE3 (ATCC) strain was used as original source for *E. coli* genes. The *ADS* from *Artemisia annua* was codon optimized for bacteria expression (Figure S3). All the genes inside each module have their own ribosome binging sites (RBS). The PAC vector was amplified from pAC-Lyc plasmid from previous study [32]. The amplified DNA fragments were purified and treated with 20 U DpnI at 37 °C for one hour. After that, 100 mM Tris-HCL at pH 9, 0.3% (v/v) iodine and 10% (v/v) ethanol were supplied to the reactions and the mixtures were heated at 70 °C for 5 min. If the mixture turned out to be colorless, additional 0.3% (v/v) iodine and 10% (v/v) ethanol would be supplied and the mixture would be heated at 70 °C for another 5 min. The DNA fragments treated with iodine and ethanol were then purified by ethanol precipitation. For CLIVA optimization experiments, 0.15 pmol of every pieces together with different kinds and concentrations of salts were heated at 80 °C for 1 min, cooled down to the temperature which was 3 degree lower than the melting temperature of the overlapped sequences, kept for 10 min and then cooled down to 20 °C at 0.1 °C/s. 0.5 µl of the assembling mixture was mixed with 50 µl of XL10-Gold competent cell for electroporation. For DXP pathway assembling experiments, all the DNA fragments were prepared at 0.25 µM and equal amount of every pieces were mixed with MgCl_2_ at 2.5 mM. The mixture were heated at 80 °C for 1 min, cooled down to 68 °C, kept for 10min and then cooled down to 20 °C at 0.1 °C/s. 0.5 µl of the assembling mixture was mixed with 50 µl of XL10-Gold competent cell for electroporation.

### Metabolite measurement

Amorphadiene was trapped in the dodecane phase and quantified as previously described [47]. The dodecane phase was diluted 100 times in ethyl acetate and the amorphadiene was quantified by Agilent 7890 gas chromatography/mass spectrometry (GC/MS) by scanning 189 and 204 m/z ions, using trans-caryophyllene as standard. The amorphadiene concentrations were adjusted to the volume of cell suspension (0.8 ml) for report.

The DXP pathway intermediates (DXP, MEP, CPD-ME, CDP-MEP, MEC, HMBPP, IPP, DMAPP, GPP, FPP, Figure 1A) were quantified by UPLC-MS as described [31]. For extracellular metabolites, the growth medium was diluted 30 times in methanol, shaken at room temperature for 2 min and centrifuged at 20,000 g for 5 min to yield the supernatant as the sample for injection. For intracellular metabolites, 1 ml x OD600 cell was collected and the medium was removed with centrifugation. The cell pellet was then suspended in 30 µl of water, 120 µl of methanol was added afterwards and the mixture was shaken at room temperature for 10 min to lyse the cells and release the intermediates [48]. The cell debris was removed by centrifugation at 20,000 g for 5 min. 5 µl of either extracellular or intracellular sample was injected. Aqueous solution containing 15 mM acetic acid and 10 mM tributylamine and methanol were used as mobile phase with a UPLC C18 column (Waters CSH C18 1.7 μm 2.1x 50 mm). The elution was done at 0.15 mL/min with gradient. A standard curve following the same treatment was used to quantify the extracellular or intracellular metabolites. The detection limit was at least 5 µM in the final sample for FPP, CDP-MEP and at least 1 µM in the final sample for the rest of the metabolites. 

## Supporting Information

Figure S1 **Different cations’ effects on the assembly efficiency.** The assembling efficiencies of PAC-SIDF plasmid with O36-38/4-5 design (36-38 bases overlap with phosphorothioate modification at each 4-5 bases) at 2.5 mM or 12.5 mM of MgCl_2_, CaCl_2_, CoCl_2_ or CuCl_2_ were presented. All the experiments were done at triplicates and the standard error were presented in the figure.
(DOC)

Figure S2 **The assembly efficiency of overlap designs with single phosphorothioate modification.** O12-13/12-13, O24-25/24-25, O36-38/36-38: 12-13 bases, 24-25 bases, 36-38 bases homologous sequences with one phosphorothioate modification. All the experiments were done at triplicates and the standard error were presented in the figure.
(DOC)

Figure S3 **the sequence of codon optimized *ADS* gene.**
(DOC)

Table S1 **Primers used for CLIVA optimization.** The phosphorothioate modifications were presented as *. The PAC-F, PAC-R, siDF-F and siDF-R were the gene specific sequences. An “Ox/y” designation was used to define the primers, where O denoted overlap; x was the length of overlap which had one modification at each y base pairs of the sequence. For example, O13/1 was a primer with 13 bases of overlap and phosphorothioate modifications at every base-pair. Similarly, O13/4 denoted a primer with 13 overlaps and phosphorothioate modifications at every 4^th^ base-pair.
(DOC)

Table S2 **Primers used for DXP pathway construction.** The phosphorothioate modifications were presented as *. And the underlined sequences were the gene specific sequences of the primers.
(DOC)

Table S3 **Design details for DXP pathway construction.**
(DOC)

Table S4 **Primers used to check the constructions with quantitative colony PCR.**
(DOC)
