# Supplementary material for: Combinatorial Engineering of 1-Deoxy-D-Xylulose 5-Phosphate Pathway Using Cross-Lapping In Vitro Assembly (CLIVA) Method
Source: PLoS One. 2013 Nov 5;8(11):e79557. doi: 10.1371/journal.pone.0079557 (PMC3818232; doi:10.1371/journal.pone.0079557)
Supplement: Figure S1 — Different cations’ effects on the assembly efficiency. The assembling efficiencies of PAC-SIDF plasmid with O36-38/4-5 design (36-38 bases overlap with phosphorothioate modification at each 4-5 bases) at 2.5 mM or 12.5 mM of MgCl2, CaCl2, CoCl2 or CuCl2 were presented. All the experiments were done at triplicates and the standard error were presented in the figure. (DOC) [file pone.0079557.s001.doc]

Figure S1. Different cations’ effects on the assembly efficiency. The assembling efficiencies of PAC-SIDF plasmid with O36-38/4-5 design (36-38 bases overlap with phosphorothioate modification at each 4-5 bases) at 2.5 mM or 12.5 mM of MgCl_2_, CaCl_2_, CoCl_2_ or CuCl_2_ were presented. All the experiments were done at triplicates and the standard error were presented in the figure.
