# Supplementary material for: Combinatorial Engineering of 1-Deoxy-D-Xylulose 5-Phosphate Pathway Using Cross-Lapping In Vitro Assembly (CLIVA) Method
Source: PLoS One. 2013 Nov 5;8(11):e79557. doi: 10.1371/journal.pone.0079557 (PMC3818232; doi:10.1371/journal.pone.0079557)
Supplement: Figure S2 — The assembly efficiency of overlap designs with single phosphorothioate modification. O12-13/12-13, O24-25/24-25, O36-38/36-38: 12-13 bases, 24-25 bases, 36-38 bases homologous sequences with one phosphorothioate modification. All the experiments were done at triplicates and the standard error were presented in the figure. (DOC) [file pone.0079557.s002.doc]

Figure S2: The assembly efficiency of overlap designs with single phosphorothioate modification. O12-13/12-13, O24-25/24-25, O36-38/36-38: 12-13 bases, 24-25 bases, 36-38 bases homologous sequences with one phosphorothioate modification. All the experiments were done at triplicates and the standard error were presented in the figure.
