# Supplementary material for: Combinatorial Engineering of 1-Deoxy-D-Xylulose 5-Phosphate Pathway Using Cross-Lapping In Vitro Assembly (CLIVA) Method
Source: PLoS One. 2013 Nov 5;8(11):e79557. doi: 10.1371/journal.pone.0079557 (PMC3818232; doi:10.1371/journal.pone.0079557)
Supplement: Figure S3 — the sequence of codon optimized ADS gene. (DOC) [file pone.0079557.s003.doc]

atgtctctgactgaggaaaaaccaatccgtccgatcgcaaactttccgccgagcatctggggtgatcagttcctgatctaccagaagcaggtcgagcaaggcgtagaacagatcgtgaacgacctgaaaaaggaggtacgtcagctgctgaaagaggctctggacatcccgatgaaacatgcgaacctgctgaagctgattgatgagattcagcgcctgggtatcccataccacttcgaacgtgaaattgatcacgcgctgcagtgtatttatgaaacgtatggtgataactggaacggtgaccgtagctccctgtggttccgtctgatgcgtaagcagggttattacgtgacctgcgacgtcttcaacaactacaaagacaagaacggcgcgtttaaacagagcctggcgaatgacgttgaaggcctgctggagctgtatgaagcaacctctatgcgtgttccgggcgaaatcatcctggaagatgctctgggtttcacccgttctcgcctgtctatcatgaccaaggacgcattttccactaacccggccctgttcaccgaaatccagcgtgcgctgaaacagcctctgtggaagcgtctgccgcgtatcgaggcggcacagtacatcccgttctatcagcaacaggattcccacaacaaaaccctgctgaaactggcgaaactggaatttaacctgctgcaatccctgcacaaagaagaactgtctcatgtttgcaaatggtggaaagccttcgacatcaagaaaaacgcgccgtgcctgcgcgaccgtatcgtagaatgctatttctggggcctgggctccggttatgagcctcaatactctcgtgctcgcgtattcttcaccaaagcggtagctgtgatcaccctgatcgacgatacctacgatgcttacggtacgtacgaagaactgaagatttttaccgaggctgttgaacgctggtctatcacttgcctggataccctgccggaatacatgaaaccgatctataaactgttcatggatacttataccgaaatggaagagttcctggcaaaagaaggtcgcactgatctgttcaactgtggcaaggagtttgtgaaagaattcgtccgcaacctgatggtggaggcgaagtgggcaaacgagggtcacatcccgaccaccgaagaacacgacccggtagttatcatcaccggtggcgcaaacctgctgactaccacttgctacctgggtatgtccgacatttttacgaaagaaagcgttgaatgggcagtgtctgcgcctccgctgttccgttactctggtatcctgggccgtcgcctgaacgatctgatgacccataaagccgagcaggaacgtaaacactccagcagctctctggaatcttacatgaaagaatacaacgttaacgaagagtatgcgcagaccctgatctacaaagaggttgaggatgtatggaaagacatcaaccgtgagtacctgactactaaaaacatcccacgcccgctgctgatggcagtgatttatctgtgccagtttctggaagtgcagtatgcgggcaaagataacttcacccgtatgggtgacgaatacaaacacctgatcaagtccctgctggtttacccaatgtccatc

Figure S3: the sequence of codon optimized *ADS* gene
