# Supplementary material for: Combinatorial Engineering of 1-Deoxy-D-Xylulose 5-Phosphate Pathway Using Cross-Lapping In Vitro Assembly (CLIVA) Method
Source: PLoS One. 2013 Nov 5;8(11):e79557. doi: 10.1371/journal.pone.0079557 (PMC3818232; doi:10.1371/journal.pone.0079557)
Supplement: Table S1 — Primers used for CLIVA optimization. The phosphorothioate modifications were presented as *. The PAC-F, PAC-R, siDF-F and siDF-R were the gene specific sequences. An “Ox/y” designation was used to define the primers, where O denoted overlap; x was the length of overlap which had one modification at each y base pairs of the sequence. For example, O13/1 was a primer with 13 bases of overlap and phosphorothioate modifications at every base-pair. Similarly, O13/4 denoted a primer with 13 overlaps and phosphorothioate modifications at every 4th base-pair. (DOC) [file pone.0079557.s004.doc]

Table S1: Primers used for CLIVA optimization

| Design | Primer  Name | Cross lapping primer | Sequence |
| --- | --- | --- | --- |
|  | PAC-F |  | GGACAGAGAGTGGAACCAACCG |
|  | PAC-R |  | GCCAAGTAGCGAAGCGAGCAG |
|  | siDF-F |  | TGCGACTCCTGCATTAGGAAGC |
|  | siDF-R |  | TCCCCGAAAAGTGCCACCTG |
| O12-13/1 | O13/1-PAC-F | O12/1-siDF-R | T*C*T*G*T*C*C*T*C*C*C*C*GAAAAGTGCCACCTG |
|  | O12/1-PAC-R | O12/1-siDF-F | A*G*T*C*G*C*A*G*C*C*A*A*G*TAGCGAAGCGAGCAG |
|  | O13/1-siDF-F | O13/1-PAC-R | C*T*T*G*G*C*T*G*C*G*A*C*T*CCTGCATTAGGAAGC |
|  | O12/1-siDF-R | O13/1-PAC-F | G*G*G*G*A*G*G*A*C*A*G*A*GAGTGGAACCAACCG |
| O12-13/4-5 | O13/4-5-PAC-F | O12/4-5-siDF-R | TCTGT*CCTC*CCC*GAAAAGTGCCACCTG |
|  | O12/4-5-PAC-R | O13/4-5-siDF-F | AGTCG*CAGC*CAAG*TAGCGAAGCGAGCAG |
|  | O13/4-5-siDF-F | O12/4-5-PAC-R | CTTGG*CTGC*GACT*CCTGCATTAGGAAGC |
|  | O12/4-5-siDF-R | O13/4-5-PAC-F | GGGG*AGGA*CAGA*GAGTGGAACCAACCG |
| O12-13/6-7 | O13/6-7-PAC-F | O12/6-7-siDF-R | TCTGTCC*TCCCC*GAAAAGTGCCACCTG |
|  | O12/6-7-PAC-R | O13/6-7-siDF-F | AGTCGCA*GCCAAG*TAGCGAAGCGAGCAG |
|  | O13/6-7-siDF-F | O12/6-7-PAC-R | CTTGGCT*GCGACT*CCTGCATTAGGAAGC |
|  | O12/6-7-siDF-R | O13/6-7-PAC-F | GGGGAG*GACAGA*GAGTGGAACCAACCG |
| O12-13/12-13 | O13/13-PAC-F | O12/12-siDF-R | TCTGTCCTCCCC*GAAAAGTGCCACCTG |
|  | O12/12-PAC-R | O13/13-siDF-F | AGTCGCAGCCAAG*TAGCGAAGCGAGCAG |
|  | O13/13-siDF-F | O12/12-PAC-R | CTTGGCTGCGACT*CCTGCATTAGGAAGC |
|  | O12/12-siDF-R | O13/13-PAC-F | GGGGAGGACAGA*GAGTGGAACCAACCG |
| O24-25/4-5 | O24/4-5-PAC-F | O24/4-5-siDF-R | CCAC*TCTC*TGTC*CTCC*CCGA*AAAG*TGCCACCTG |
|  | O25/4-5-PAC-R | O25/4-5-siDF-F | TGCAG*GAGT*CGCA*GCCA*AGTA*GCGA*AGCGAGCAG |
|  | O25/4-5-siDF-F | O25/4-5-PAC-R | TCGC*TACTT*GGCT*GCGA*CTCC*TGCA*TTAGGAAGC |
|  | O24/4-5-siDF-R | O24/4-5-PAC-F | CTTT*TCGG*GGAG*GACA*GAGA*GTGG*AACCAACCG |
| O24-25/24-25 | O24/24-PAC-F | O24/24-siDF-R | CCACTCTCTGTCCTCCCCGAAAAG*TGCCACCTG |
|  | O25/25-PAC-R | O25/25-siDF-F | TGCAGGAGTCGCAGCCAAGTAGCGA*AGCGAGCAG |
|  | O25/25-siDF-F | O25/25-PAC-R | TCGCTACTTGGCTGCGACTCCTGCA*TTAGGAAGC |
|  | O24/24-siDF-R | O24/24-PAC-F | CTTTTCGGGGAGGACAGAGAGTGG*AACCAACCG |
| O36-38/4-5 | O38/4-5-PAC-F | O38/4-5-siDF-R | GTGG*CACTTT*TCGG*GGAG*GACAG*AGAGT*GGAA*CCAA*CCG |
|  | O36/4-5-PAC-R | O36/4-5-siDF-F | TCCTAA*TGCAG*GAGTC*GCAG*CCAA*GTAGC*GAAG*CGAG*CAG |
|  | O36/4-5-siDF-F | O36/4-5-PAC-R | CTCG*CTTCG*CTACT*TGGCT*GCGA*CTCCT*GCATT*AGGA*AGC |
|  | O38/4-5-siDF-R | O38/4-5-PAC-F | TTGGTT*CCAC*TCTCT*GTCC*TCCC*CGAAA*AGTG*CCAC*CTG |
| O38/36-38 | O38/38-PAC-F | O38/38-siDF-R | GTGGCACTTTTCGGGGAGGACAGAGAGTGGAACCAA*CCG |
|  | O36/36-PAC-R | O36/36-siDF-F | TCCTAATGCAGGAGTCGCAGCCAAGTAGCGAAGCGAG*CAG |
|  | O36/36-siDF-F | O36/36-PAC-R | CTCGCTTCGCTACTTGGCTGCGACTCCTGCATTAGGA*AGC |
|  | O38/38-siDF-R | O38/38-PAC-F | TTGGTTCCACTCTCTGTCCTCCCCGAAAAGTGCCAC*CTG |

The phosphorothioate modifications were presented as *. The PAC-F, PAC-R, siDF-F and siDF-R were the gene specific sequences. An “Ox/y” designation was used to define the primers, where O denoted overlap; x was the length of overlap which had one modification at each y base pairs of the sequence. For example, O13/1 was a primer with 13 bases of overlap and phosphorothioate modifications at every base-pair. Similarly, O13/4 denoted a primer with 13 overlaps and phosphorothioate modifications at every 4^th^ base-pair.
