# Supplementary material for: Combinatorial Engineering of 1-Deoxy-D-Xylulose 5-Phosphate Pathway Using Cross-Lapping In Vitro Assembly (CLIVA) Method
Source: PLoS One. 2013 Nov 5;8(11):e79557. doi: 10.1371/journal.pone.0079557 (PMC3818232; doi:10.1371/journal.pone.0079557)
Supplement: Table S2 — Primers used for DXP pathway construction. The phosphorothioate modifications were presented as *. And the underlined sequences were the gene specific sequences of the primers. (DOC) [file pone.0079557.s005.doc]

Table S2: Primers used for DXP pathway construction

| Name | Cross lapping primer | Sequence |
| --- | --- | --- |
| CL-pET-1F | CL-pAC-R | CTCG*CTTCG*CTACT*TGGCT*GCGA*CTCCT*GCATT*AGGA*AGC |
| CL-pET-2F | CL-pET-aR | CCGC*AAGAG*GCCC*GCAGT*AGTAG*GTTGA*GGCC*GTTGA |
| CL-pET-3F | CL-pET-bR | GTACC*GGCA*TAACC*AAGCC*ACCG*CCGC*CGC*AAGG*AAT |
| CL-pET-4F | CL-pET-cR | CTACA*GCATC*CAGG*GTGA*CCCT*GCCA*CCATA*CCCA*CGC |
| CL-pET-5F | CL-pET-dR | CGAG*GATGA*CGATG*AGCG*TGAGC*CCGA*AGTG*GCG*AGC |
| CL-pET-6F | CL-pET-eR | CTGAC*TGCG*TTAGC*AATTTA*ACAGC*AACC*GCAC*CTGT*GGC |
| CL-pET-7F | CL-pET-fR | AGAC*GAAAG*GGCC*TCGG*ATGC*GTCC*GGCG*TAGA*GGA |
| CL-pAC-F | CL-pET-gR | GTGG*CACTTT*TCGG*GGAG*GACAG*AGAGT*GGAA*CCAA*CCG |
| CL-pAC-R | CL-pET-1F | TCCTAA*TGCAG*GAGTC*GCAG*CCAA*GTAGC*GAAG*CGAG*CAG |
| CL-pET-aR | CL-pET-2F | GGCC*TCAAC*CTACT*ACTGC*GGGC*CTCTT*GCGG*GATA |
| CL-pET-bR | CL-pET-3F | CCTTG*CGGC*GGCG*GTGG*CTTG*GTTAT*GCCG*GTAC*TGC |
| CL-pET-cR | CL-pET-4F | TGGG*TATGG*TGGC*AGGG*TCACC*CTGGA*TGCT*GTAG*GCA |
| CL-pET-dR | CL-pET-5F | CGCC*ACTTC*GGGC*TCACG*CTCA*TCGT*CATC*CTCG*GCA |
| CL-pET-eR | CL-pET-6F | ACAGG*TGCG*GTTGC*TGTTA*AATTG*CTAAC*GCAG*TCAG*GCA |
| CL-pET-fR | CL-pET-7F | TCTACG*CCGG*ACGCA*TCCG*AGGC*CCTTT*CGTCT*TCA |
| CL-pET-gR | CL-pAC-F | TTGGTT*CCAC*TCTCT*GTCC*TCCC*CGAAA*AGTG*CCAC*CTG |

The phosphorothioate modifications were presented as *. And the underlined sequences were the gene specific sequences of the primers.
