# Supplementary material for: Combinatorial Engineering of 1-Deoxy-D-Xylulose 5-Phosphate Pathway Using Cross-Lapping In Vitro Assembly (CLIVA) Method
Source: PLoS One. 2013 Nov 5;8(11):e79557. doi: 10.1371/journal.pone.0079557 (PMC3818232; doi:10.1371/journal.pone.0079557)
Supplement: Table S3 — Design details for DXP pathway construction. (DOC) [file pone.0079557.s006.doc]

Table S3: Design details for DXP pathway construction.

|  | **Modules** | | | | | | | | |
| --- | --- | --- | --- | --- | --- | --- | --- | --- | --- |
| Symbol | S | R | DEF | GH | IAA | ISC | SUF | PAC | IAA-PAC |
| Genes | T7-dxs | T7-dxr | T7-ispE-ispD-ispF | T7-ispG-ispH | T7-ADS-ispA-idi | T7-iscS-iscU-iscA-hscB-hscA-fdx | T7-sufA-sufB-sufC-sufD-sufS-sufE | PAC | T7-ADS-ispA-idi-PAC |
| Template | pET-dxs | pET-dxr | pET-DEF | pET-GH | pET-IAA | pET-ISC | pET-SUF | pAC-lyc | IAA-PAC |
| **Plasmids** | **Primers for used to amplify the modules** | | | | | | | | |
| IAA-PAC | - | - | - | - | CL-pET-1F  CL-pET-gR | - | - | CL-PAC-F  CL-PAC-R | - |
| S-IAA-PAC | CL-pET-1F  CL-pET-fR | - | - | - | - | - | - | - | CL-pET-7F  CL-PAC-R |
| S-R-IAA-PAC | CL-pET-1F  CL-pET-aR | CL-pET-2F  CL-pET-fR | - | - | - | - | - | - | CL-pET-7F  CL-PAC-R |
| S-DEF-IAA-PAC | CL-pET-1F  CL-pET-bR | - | CL-pET-3F  CL-pET-fR | - | - | - | - | - | CL-pET-7F  CL-PAC-R |
| S-GH-IAA-PAC | CL-pET-1F  CL-pET-cR | - | - | CL-pET-4F  CL-pET-fR | - | - | - | - | CL-pET-7F  CL-PAC-R |
| S-R-DEF-IAA-PAC | CL-pET-1F  CL-pET-aR | CL-pET-2F  CL-pET-bR | CL-pET-3F  CL-pET-fR | - | - | - | - | - | CL-pET-7F  CL-PAC-R |
| S-R-GH-IAA-PAC | CL-pET-1F  CL-pET-bR | - | CL-pET-3F  CL-pET-cR | CL-pET-4F  CL-pET-fR | - | - | - | - | CL-pET-7F  CL-PAC-R |
| S-DEF-GH-IAA-PAC | CL-pET-1F  CL-pET-aR | CL-pET-2F  CL-pET-cR | - | CL-pET-4F  CL-pET-fR | - | - | - | - | CL-pET-7F  CL-PAC-R |
| S-R-DEF-GH-IAA-PAC | CL-pET-1F  CL-pET-aR | CL-pET-2F  CL-pET-bR | CL-pET-3F  CL-pET-cR | CL-pET-4F  CL-pET-fR | - | - | - | - | CL-pET-7F  CL-PAC-R |
| S-ISC-IAA-PAC | CL-pET-1F  CL-pET-dR | - | - | - | - | CL-pET-5F  CL-pET-fR | - | - | CL-pET-7F  CL-PAC-R |
| S-SUR-IAA-PAC | CL-pET-1F  CL-pET-eR | - | - | - | - | - | CL-pET-6F  CL-pET-fR | - | CL-pET-7F  CL-PAC-R |
| S-GH-ISC-IAA-PAC | CL-pET-1F  CL-pET-cR | - | - | CL-pET-4F  CL-pET-dR | - | CL-pET-5F  CL-pET-fR | - | - | CL-pET-7F  CL-PAC-R |
| S-GH-SUR-IAA-PAC | CL-pET-1F  CL-pET-cR | - | - | CL-pET-4F  CL-pET-eR | - | - | CL-pET-6F  CL-pET-fR | - | CL-pET-7F  CL-PAC-R |
| S-R-GH-ISC-IAA-PAC | CL-pET-1F  CL-pET-bR | - | CL-pET-3F  CL-pET-cR | CL-pET-4F  CL-pET-dR | - | CL-pET-5F  CL-pET-fR | - | - | CL-pET-7F  CL-PAC-R |
| S-R-GH-SUR-IAA-PAC | CL-pET-1F  CL-pET-bR | - | CL-pET-3F  CL-pET-cR | CL-pET-4F  CL-pET-eR | - | - | CL-pET-6F  CL-pET-fR | - | CL-pET-7F  CL-PAC-R |
| S-R-DEF-GH-ISC-IAA-PAC | CL-pET-1F  CL-pET-aR | CL-pET-2F  CL-pET-bR | CL-pET-3F  CL-pET-cR | CL-pET-4F  CL-pET-dR | - | CL-pET-5F  CL-pET-fR | - | - | CL-pET-7F  CL-PAC-R |
