# Supplementary material for: Combinatorial Engineering of 1-Deoxy-D-Xylulose 5-Phosphate Pathway Using Cross-Lapping In Vitro Assembly (CLIVA) Method
Source: PLoS One. 2013 Nov 5;8(11):e79557. doi: 10.1371/journal.pone.0079557 (PMC3818232; doi:10.1371/journal.pone.0079557)
Supplement: Table S4 — Primers used to check the constructions with quantitative colony PCR. (DOC) [file pone.0079557.s007.doc]

Table S4: Primers used to check the constructions with quantitative colony PCR

| Name | Position | Sequence |
| --- | --- | --- |
| dxs-1609F | S, sense | CCGCTTGATGAAGCGTTAATTCTGG |
| dxs-122R | S, antisense | GGAACGGCTCACGCTGT |
| dxr-704F | R, sense | AAGGTCTGGAATACATTGAAGC |
| dxr-782R | R, antisense | CACTGCCGTCCTGATAGC |
| ispF-220F | DEF, sense | TTAAAGGTGCCGATAGCC |
| ispE-349R | DEF, antisense | ATTGCCAGAGATGATTTAATGC |
| ispH-693F | GH, sense | CTCCAACTCCAACCGTCTG |
| ispG-329R | GH, antisense | ACGCTCTTCATTACCGATATTGC |
| idi-462F | IAA, sense | TGTATTACACGGTATTGATGCCACG |
| ADS-941R | IAA, antisense | GCTTTGGTGAAGAATACGCGAGCA |
| PAC-seqF | PAC, sense | CCTGCTCGCTTCGCTACT |
| PAC-seqR | PAC, antisense | GCGGTGCGGACTGTTG |
| FDX-89F | ISC, sense | CTCTGCGTAACGGTATCG |
| iscS-601R | ISC, antisense | ACATCAGGTCAACTTTCAACT |
| surfA-334R | SUF, sense | TCTGGGCTTTAGGGTTGT |
| surfE-273F | SUF, antisense | GATGACGCCGCAGGATAT |
